# Editorial: Genes, cells, and macroenvironments: regulating the immune response in extreme conditions

**DOI:** 10.3389/fimmu.2025.1674347

**Published:** 2025-08-25

**Authors:** Giuseppina Pennesi

**Affiliations:** Azienda Sanitaria Territoriale Fermo, Fermo, Italy

**Keywords:** immunoregulation, genes, environmental factor, extreme environment, high altitude, microgravity, mechanotransduction, heat stress

An organism living in extreme conditions is living in a habitat that is considered very hard to survive due to its considerably severity such as very high or low gravity and mechanical loading, high or low content of oxygen or carbon dioxide in the atmosphere, high levels of radiation, extremely hot or cold weather, isolation. Adaptation to these environments requires the remodeling of diverse physiological processes, and the subsequent creation the of a new homeostasis (1, 2). The immune system, being the body’s defense mechanism against foreign organisms or substances, can be pivotal in this adaptation process, with both its innate and adaptive response; its regulation depends upon a fine network of genes activated in response to organic or environmental stresses.

Hypobaric hypoxia found at high altitude is the key factor breaking the organism’s homeostasis, including the immune response. Pivotal modifying mechanisms include molecular responses, most notably hypoxia inducible factors (HIF), with effects on mitochondrial functions and REDOX homeostasis. The outcome of HIF activation reverberates on the activation of an inflammation process followed by an innate immune response (3).

Microgravity severely affects the immune system. Since the beginning of the space mission era, studies have shown that astronauts returning from space have a compromised immune system, being prone to bacterial and viral infections shortly after returning to earth. The effects of microgravity could be due to the lack of gravitational force on the cells impairing the mechanotransduction process, that is the mechanism by which the cells convert mechanical forces into biochemical signals that then induce downstream pathways. It has been shown that mechanotransduction affects the expression of immune-related genes through the activation of cell/matrix pathways (4).

Thermoneutrality, defined as the metabolic state of an organism in an environmental temperature at which it does not have to generate or lose heat, is important for maintaining the immune homeostasis; its impairment can affect both innate and adaptive immune response (5). Heat stress, such it occurs in period of extremely hot weather, induces the production of endotoxins stimulating massive innate immune activation with increased levels of IL-6, TNF-α, and IL-1. In parallel, anti-inflammatory pathways are also activated, with the production of IL-1Ra, IL-10, and soluble TNF receptors together with the production of heat shock proteins; these anti-inflammatory pathways, however, are often not able to outweigh the pro-inflammatory ones (6).

The interplay of genes and environmental factors in regulating the immune response in extreme conditions is still not completely clarified. A better understanding of these mechanism will help not only the knowledge of the pathophysiology of the immune system, but also the quest for the treatment of immune-mediated conditions.
